# Molecular characterization and antimicrobial resistance of potentially human‐pathogenic *Escherichia coli* strains isolated from riding horses

**DOI:** 10.1186/s12917-021-02832-x

**Published:** 2021-03-25

**Authors:** Pouya Reshadi, Fatemeh Heydari, Reza Ghanbarpour, Mahboube Bagheri, Maziar Jajarmi, Mohadese Amiri, Hesam Alizade, Mahdi Askari Badouei, Shademan Sahraei, Nasrin Adib

**Affiliations:** 1 Department of Pathobiology, Faculty of Veterinary Medicine, Shahid Bahonar University of Kerman, Kerman, Iran; 2 Molecular Microbiology Research Group, Shahid Bahonar University of Kerman, Kerman, Iran; 3 Department of Food Science and Technology, Bardsir Faculty of Agriculture, Shahid Bahonar University of Kerman, Kerman, Iran; 4 Mashhad Branch, Razi Vaccine and Serum Research Institute, Agricultural Research, Education and Extension Organization (AREEO), Mashhad, Iran; 5 Infectious and Tropical Diseases Research Center, Hormozgan Health Institute, Hormozgan University of Medical Sciences, Bandar Abbas, Iran; 6 Department of Pathobiology, Faculty of Veterinary Medicine, Ferdowsi University of Mashhad, Mashhad, Iran

**Keywords:** *Escherichia coli*, Antimicrobial resistance, Virulence factors, Phylogeny, Horse

## Abstract

**Background:**

Transmission of antimicrobial resistant and virulent *Escherichia coli* (*E. coli*) from animal to human has been considered as a public health concern. This study aimed to determine the phylogenetic background and prevalence of diarrheagenic *E. coli* and antimicrobial resistance in healthy riding-horses in Iran. In this research, the genes related to six main pathotypes of *E. coli* were screened. Also, genotypic and phenotypic antimicrobial resistance against commonly used antibiotics were studied, then phylo-grouping was performed on all the isolates.

**Results:**

Out of 65 analyzed isolates, 29.23 % (*n* = 19) were determined as STEC and 6.15 % (*n* = 4) as potential EPEC. The most prevalent antimicrobial resistance phenotypes were against amoxicillin/clavulanic acid (46.2 %) and ceftriaxone (38.5 %). *bla*_*TEM*_ was the most detected resistance gene (98.4 %) among the isolates and 26.15 % of the *E. coli* isolates were determined as multi-drug resistant (MDR). Three phylo-types including B1 (76.92 %), A (13.85 %) and D (3.08 %) were detected among the isolates.

**Conclusions:**

Due to the close interaction of horses and humans, these findings would place emphasis on the pathogenic and zoonotic potential of the equine strains and may help to design antimicrobial resistance stewardship programs to control the dissemination of virulent and multi-drug resistant *E. coli* strains in the community.

## Background

Over time, horses have been bred by humans for meat, leisure and sport. Today, horses are known as important companion animals and have become popular for horse-back riding. Horse-riding clubs are a significant platform for human-horse interaction due to close contact between horses, horse handlers, horse riders and spectators [1]. Horses as companion animals could be considered as a potential reservoir of microbial agents which cause infections and complications in various hosts such as human. Among these microorganisms, some strains of *Escherichia coli* (*E. coli*) possess antimicrobial resistance (AMR) and virulence determinants which could be transmitted by direct or indirect contact [2].

A major group of *E. coli* strains, designated as diarrheagenic *E. coli* (DEC), cause intestinal infections [3]. According to the pathogenesis of DECs, they are divided into six main pathotypes including enterotoxigenic *E. coli* (ETEC), enteropathogenic *E. coli* (EPEC), enteroinvasive *E. coli* (EIEC), enteroaggregative *E. coli* (EAEC), diffusely adherent *E. coli* (DAEC) and Shiga toxin-producing *E. coli* (STEC) containing a sub-pathotype named enterohemorrhagic *E. coli* (EHEC) [4]. Animals are usually considered as asymptomatic source of DECs, shedding these strains to the environment.

Many strains of *E. coli* have intrinsic or/and acquired antimicrobial resistance which should be addressed as a significant threat to public health. Resistant *E. coli* could be selected among gut microbiota due to use of antimicrobial agents. Epidemiologically, antimicrobial resistant *E. coli* strains and their AMR determinants may be transferred from animal to human by horizontal transmission of AMR genes or clonal transfer of resistant strains via direct contact, indirect contact and consumption of fecal contaminated food.

Based on phylogenetic assessments, *E. coli* have been classified into eight phylo-groups including A, B1, B2, C, D, E, F and *Escherichia* cryptic clade I, by a PCR method [5]. The gut commensal *E. coli* strains predominantly belong to group A or B1, however, antimicrobial susceptible strains usually belong to the B1 rather than groups A and D [6, 7]. *E. coli* phylogenetic background is of importance for understanding the relationship between strains, antimicrobial resistance and disease [8].

To the best of our knowledge, there are no comprehensive study on virulence, antimicrobial resistance and phylogenetic analysis of equine *E. coli* isolates in Middle East. Hence, this study was designed to assess these variables in healthy riding horses in Iran to assess the potential risks of these animals for public health. The results could help to understand the public health risks associated with horse-riding clubs and transmission of antimicrobial resistance and virulence factors of *E. coli* from horses to humans.

## Methods

### Sampling and ***E. coli*** isolation

In this study, 65 rectal swabs were collected from healthy horses (n = 65) from five riding horse clubs during July to September, 2018, in Kerman province, southeast of Iran. Sterile saline moistened swabs were inserted 10 cm into the rectum and carried to veterinary microbiology laboratory in individual tubes containing Amies transport Medium (Merck, Germany) within 12 h. For *E. coli* isolation, all samples were cultured on MacConkey agar (Merck, Germany) and incubated at 37**°**C for 24 h. One presumptive *E. coli* colony was selected from each sample and confirmed via biochemical tests. One confirmed *E. coli* isolate from each sample was subjected to phenotypic resistance analysis and genetic assessments.

### Phenotypic antimicrobial resistance assessment

In this study we evaluated antimicrobial resistance of isolates to seven antimicrobial agents which are the drugs of choice in the treatment of equine bacterial infections by disk diffusion method; including amoxicillin/clavulanic acid (20/10 µg), cefazolin (30 µg), ceftriaxone (30 µg), amikacin (30 µg), streptomycin (10 µg), gentamicin (10 µg) and trimethoprim/sulphamethoxazole (1.25/23.7 µg). The horse-specific/human breakpoints have been used to evaluate the results of disk diffusion method; the diameter of growth inhibition zones have been measured and the *E. coli* isolates were determined as resistant, intermediate and susceptible groups. According to CLSI VET08, human breakpoints are considered to provide zones of inhibition when there are no veterinary breakpoints available for some antimicrobial agents for all animal species [9, 10]. *E. coli* ATCC 25922 was used for quality control of the test (Table 1).

**Table 1:** Zone diameter interpretive criteria (mm) by disk diffusion method for *E. coli*

Antimicrobial agents (Disk Content)	Abbreviations	Susceptible	Intermediate	Resistant	References
Amoxicillin/clavulanic acid (20/10 µg)	AMC	≥ 18	14–17	≤ 13	[9]
Cefazolin (30 µg)	CZ	≥ 23	20–22	≤ 19	[9]
Ceftriaxone (30 µg)	CRO	≥ 23	20–22	≤ 19	[9]
Amikacin (30 µg)	AK	≥ 17	15–16	≤ 14	[9]
Streptomycin (10 µg)	S	≥ 15	12–14	≤ 11	[9]
Gentamicin (10 µg)	GN	≥ 16	13–15	≤ 12	[10]
Trimethoprim-sulphamethoxazole (1.25/23.7 µg)	SXT	≥ 16	11–15	≤ 10	[9]

### PCR for antimicrobial resistance, virulence and phylogenetic sequences

Boiling method was used for DNA extraction; a single colony was suspended in 300 µl sterile distilled water, heated up to 98 °C in heating block (Eppendorf, Germany) for 10–15 min, centrifuged in 13,000 rpm for 2 min and the supernatant was stored at -20 °C. The DNA extracts were used as templates in PCR to determine the presence or absence of antimicrobial resistance genes including *bla*_*TEM*,_*bla*_*SHV*_, *bla*_*CTX*−*M*_, *aadA*, *dhfr1*, *dhfr5*, *sul1* and *sul2* [11–15] (Table 2).

Six intestinal human pathogenic pathotypes of *E. coli* were screened by evaluation of *stx1*, *stx2*, *eae*, *stII*, *lt*, *ipaH*, *aafII* and *daaE* [16]; EPECs, EIECs, EAECs and DAECs are positive for *eae*, *ipaH*, *aafII* and *daaE*, respectively. STECs harbor *stx1* and/or *stx2* genes and ETECs are positive for *stII* and/or *lt* (Table 2).

Phylogenetic background of all isolates was determined using the PCR-based method explained by Clermont et al. (2013). In this scheme an *E. coli* strain could be classified into one of the phylo-types A, B1, B2, C, D, E, F and cryptic clades I to V [5] (Table 2).

All PCR methods were carried out as simplex in 25 µl final reaction volume including 3 µl DNA extract, 0.3 µM of each primer, 12.5 µl 2× Taq DNA Polymerase Master Mix RED (Ampliqon, Denmark) and sterile distilled water up to 25 µl. PCR products were loaded on an 1.5 % electrophoresis agarose gel containing Green Viewer stain (ParsTous, Iran) and imaged by a GelDoc 1000 (Vilber Lourmat, France).

### Statistical analysis

For descriptive statistical analysis, all data about presence or absence of studied factors in each strain were imported into SPSS (SPSS 19; IBM) program as binomial variables; prevalence, 95 % confidence level and *P* value were calculated.

**Table 2:** Primer sequences and PCR condition for detection of antimicrobial resistance and phylo-group genes

Target (subject)	Sequence (5’–3’)	PCR condition	Product size (bp)	Reference
*bla*_*TEM*_ (Resistance to β-lactam)	F^a^-GCGGAACCCCTATTTG	35 cycles: 95°C (30 s), 52°C (30 s), 72°C (60 s)	963	[11]
	R^b^-ACCAATGCTTAATCAGTGAG			
*bla*_*SHV*_ (Resistance to β-lactam)	F-TTATCTCCCTGTTAGCCACC	35 cycles: 95°C (30 s), 54°C (30 s), 72°C (30 s)	795	[12]
	R-GATTTGCTGATTTCGCTCGG			
*bla*_*CTX-M*_ (Resistance to β-lactam)	F-CGATGTGCAGTACCAGTAA	35 cycles: 95°C (30 s), 60°C (30 s), 72°C (60 s)	585	[13]
	R-TTAGTGACCAGAATCAGCGG			
*sulI* (Resistance to sulfonamide)	F-TTCGGCATTCTGAATCTCAC	30 cycles: 94°C (30 s), 58°C (30 s), 72°C (60 s)	822	[14]
	R-ATGATCTAACCCTCGGTCTC			
*sulII* (Resistance to sulfonamide)	F-GCGCTCAAGGCAGATGGCATT	30 cycles: 94°C (30 s), 69°C (30 s), 72°C (60 s)	293	[15]
	R-GCGTTTGATACCGGCACCCGT			
*dhfrI* (Resistance to trimethoprim)	F-AAGAATGGAGTTATCGGGAATG	30 cycles: 94°C (30 s), 55°C (30 s), 72°C (60 s)	391	[14]
	R-GGGTAAAAACTGGCCTAAAATTG			
*dhfrV* (Resistance to trimethoprim)	F-CTGCAAAAGCGAAAAACGG	35 cycles: 94°C (30 s), 58°C (30 s), 72°C (60 s)	432	[14]
	R-AGCAATAGTTAATGTTTGAGCTAAAG			
*aadA* (Resistance to aminoglycoside)	F-TGATTTGCTGGTTACGGTGAC	30 cycles: 94°C (30 s), 58°C (30 s), 72°C (60 s)	284	[14]
	R-CGCTATGTTCTCTTGCTTTTG			
*stx1* (STEC)	F-CAGTTAATGTGGTGGCGAAGG	35 cycles: 94°C (90 s), 60°C (90 s), 72°C (90 s)	348	[16]
	R-CACCAGACAATGTAACCGCTG			
*stx2* (STEC)	F-ATCCTATTCCCGGGAGTTTACG	35 cycles: 94°C (90 s), 60°C (90 s), 72°C (90 s)	584	[16]
	R-GCGTCATCGTATACACAGGAGC			
*eae* (EPEC)	F-TCAATGCAGTTCCGTTATCAGTT	35 cycles: 94°C (90 s), 60°C (90 s), 72°C (90 s)	482	[16]
	R-GTAAAGTCCGTTACCCCAACCTG			
*stII* (ETEC)	F-AAAGGAGAGCTTCGTCACATTTT	35 cycles: 94°C (90 s), 60°C (90 s), 72°C (90 s)	129	[16]
	R-AATGTCCGTCTTGCGTTAGGAC			
*lt* (ETEC)	F-GCACACGGAGCTCCTCAGTC	35 cycles: 94°C (90 s), 60°C (90 s), 72°C (90 s)	218	[16]
	R-TCCTTCATCCTTTCAATGGCTTT			
*ipaH* (EIEC)	F-CTCGGCACGTTTTAATAGTCTGG	35 cycles: 94°C (90 s), 60°C (90 s), 72°C (90 s)	933	[16]
	R-GTGGAGAGCTGAAGTTTCTCTGC			
*aafII* (EAEC)	F-CACAGGCAACTGAAATAAGTCTGG	35 cycles: 94°C (90 s), 60°C (90 s), 72°C (90 s)	378	[16]
	R-ATTCCCATGATGTCAAGCACTTC			
*daaE* (DAEC)	F-GAACGTTGGTTAATGTGGGGTAA	35 cycles: 94°C (90 s), 60°C (90 s), 72°C (90 s)	542	[16]
	R-TATTCACCGGTCGGTTATCAGT			
*arpA* (phylo-grouping)	F-AACGCTATTCGCCAGCTTGC	30 cycles: 94°C (5 s), 59°C (20 s) and 1 cycle 72°C (5 min)	400	[5]
	R-TCTCCCCATACCGTACGCTA			
*chuA* (phylo-grouping)	F-ATGGTACCGGACGAACCAAC	30 cycles: 94°C (5 s), 59°C (20 s) and 1 cycle 72°C (5 min)	288	[5]
	R-TGCCGCCAGTACCAAAGACA			
*yjaA* (phylo-grouping)	F-CAAACGTGAAGTGTCAGGAG	30 cycles: 94°C (5 s), 59°C (20 s) and 1 cycle 72°C (5 min)	211	[5]
	R-AATGCGTTCCTCAACCTGTG			
*TspE4.C2* (phylo-grouping)	F-CACTATTCGTAAGGTCATCC	30 cycles: 94°C (5 s), 59°C (20 s) and 1 cycle 72°C (5 min)	152	[5]
	R-AGTTTATCGCTGCGGGTCGC			
*arpA*-group E (phylo-grouping)	F-GATTCCATCTTGTCAAAATATGCC	30 cycles: 94°C (5 s), 57°C (20 s) and 1 cycle 72°C (5 min)	301	[5]
	R-GAAAAGAAAAAGAATTCCCAAGAG			
*trpA*-group C (phylo-grouping)	F-AGTTTTATGCCCAGTGCGAG	30 cycles: 94°C (5 s), 59°C (20 s) and 1 cycle 72°C (5 min)	219	[5]
	R-TCTGCGCCGGTCACGCCC			

^a^ Forward, ^b^ Reverse

## Results

In the present study, sixty five *E. coli* strains isolated from 65 horses have been evaluated. The most prevalent antimicrobial resistance phenotype was against the two β-lactam antibiotics, amoxicillin/clavulanic acid (46.2 %) and ceftriaxone (38.5 %), and the other antimicrobial resistance (AR) phenotypes were observed in less than 25 % of the *E. coli* isolates. In agreement with the phenotypic findings, the gene related to β-lactam resistance (*bla*_*TEM*_) was the most detected gene (98.4 %) among the isolates (Table 3).

Twenty-three phenotypic AR patterns were recognized in this study. No significant difference was observed in prevalence of AR patterns (*p-*value > 0.05); the frequency of the patterns was in the range of 1 to 4. According to studied antimicrobial agents, 26.15 % (n = 17) of the isolates were determined as multi-drug resistant; resistant strains to at least three antimicrobial agents from three different antimicrobial classes are defined as MDR [17]. Most of MDR isolates belonged to B1 phylo-group (12/17; 70.58 %), followed by A (3/17), Unknown (1/17) and D (1/17) phylo-types (Tables 3 and 4).

The isolates have been screened for the presence of 8 virulence genes (VGs) to determine intestinal human diarrheagenic *E. coli* pathotypes. According to the findings, 29.23 % (19/65) of our isolates were determined as STEC and 6.15 % (4/65) as potential EPEC. Among STECs (n = 19), three virulence gene (VG) profiles were observed including *stx1* (15/19; 78.94 %), *stx1*/*stx2* (2/19; 10.52 %) and *stx1/eae* (2/19; 10.52 %); the latter is similar to some human EHEC gene profiles and *stx1* was the most prevalent profile significantly (*p-*value < 0.0001). Most of VG-positives belonged to B1 phylo-group (20/23; 86.95 %), followed by A (1/23; 4.34 %), D (1/23; 4.34 %) and Unknown (1/23; 4.34 %) phylo-types (Table 5).

Among the resistant VG-positive isolates (n = 14), five isolates were phenotypically multidrug resistant. All of the VG-positives harbored at least one of screened AR genes excluding one; three different AR gene profiles were identified including *bla*_*TEM*_, *bla*_*TEM*_/*sulII*, *bla*_*TEM*_/*bla*_*CTX*−*M*_ in which *bla*_*TEM*_ was the most prevalent significantly (*p-*value *<* 0.0001). All VG^+^/AR-gene^+^ isolates belonged to B1 except one belonging to A phylo-type (Table 6).

In this study, sixty one *E. coli* isolates have been distributed among three phylo-types including B1 (76.92 %), A (13.85 %) and D (3.08 %). Four (6.15 %) *E. coli* isolates could not be classified into the phylo-groups according to Clermont scheme and named as unknown (U). Similarly, B1 was the most prevalent phylo-type in virulent/non-virulent and resistant/non-resistant isolates; no significant difference (*p-*value > 0.05) has been observed in phylogenic distribution patterns of virulent and resistant isolates (Tables 3 and 4).

**Table 3:** Prevalence of antimicrobial resistance phenotype and genotype among *E. coli* isolates

Antimicrobial classes	AR phenotype	Number	Percentage	95% CI	AR gene	Number	Percentage	95% CI
β-lactams (Penicillin and cephalosporins)	AMC	30/65	46.2	±11.5	*bla*_*TEM*_	64/65	98.4	±1.2
	CRO	25/65	38.5	±12.1	*bla*_*SHV*_	0/65	-	-
	CZ	12/65	18.5	±11	*bla*_*CTX-M*_	4/65	6.1	±8.6
Aminoglycoside	AK	16/65	24.6	±11.6	*aadA*	0/65	-	-
	S	14/65	21.5	±11.4				
	GN	11/65	16.9	±10.9				
Sulfonamide/trimethoprim	SXT	9/65	13.8	±10.4	*sulI*	1/65	1.5	±6.6
					*sulII*	7/65	10.7	±9.8
					*dhfrI*	1/65	1.5	±6.6
					*dhfrV*	0/65	-	-

*AR* antimicrobial resistance; *CI* confidence interval; *AMC* amoxicillin/clavulanic acid; *CRO* ceftriaxone ; *CZ* cefazolin; *AK* amikacin; *S* streptomycin; *GN* gentamicin; *SXT* trimethoprim/sulphamethoxazole; antimicrobial resistance genes are abbreviated and Italic

**Table 4:** Phenotypic antimicrobial resistance profiles and their distribution pattern among phylo-groups

Order	Antimicrobial resistance profiles	Phylo-group (no.)	Total
1	GN	B1(1)	1
2	CRO	B1(4)	4
3	AMC	B1(3)	3
4	AK, S	B1(2)	2
5	GN, CRO	B1(1)	1
6	GN, AMC	B1(1)	1
7	SXT, AMC	B1(2), U(1)	3
8	CRO, AMC	A(1), B1(3)	4
9	SXT, AMC, S^a^	B1(1)	1
10	CRO, AK, CZ	A(1)	1
11	AMC, AK, CZ	B1(2)	2
12	CRO, AMC, CZ	B1(1)	1
13	CRO, AMC, AK^a^	B1(2), U(1)	3
14	GN, CRO, CZ, S^a^	A(1)	1
15	SXT, GN, AMC, S^a^	A(2)	2
16	SXT, CRO, AMC, S^a^	B1(1)	1
17	CRO, AMC, AK, CZ^a^	B1(1)	1
18	GN, CRO, AMC, AK^a^	B1(1)	1
19	CRO, AMC, AK, CZ, S^a^	B1(2)	2
20	GN, CRO, AMC, AK, S^a^	B1(1)	1
21	GN, CRO, AMC, AK, CZ, S^a^	B1(2)	2
22	SXT, GN, CRO, AMC, CZ, S^a^	D(1)	1
23	SXT, CRO, AMC, AK, CZ, S^a^	B1(1)	1
24	Non-resistant isolates	A(4), B1(18), D(1), U(2)	25
25	Total	A(9), B1(50), D(2), U(4)	65

^a^Multi-drug resistant profiles

**Table 5:** Distribution pattern of AR (antimicrobial resistance) and virulence gene profiles among phylo-groups

Profiles	A	B1	D	U^a^	Total
AR gene profiles					
* bla*_*TEM*_	6	43	1	3	53
* bla*_*TEM*_*/sulII*	-	4	-	1	5
* bla*_*TEM*_*/dhfrI*	-	1	-	-	1
* bla*_*TEM*_*/bla*_*CTX−M*_	1	2	-	-	3
* bla*_*TEM*_*/sulI/sulII*	1	-	-	-	1
* bla*_*TEM*_*/bla*_*CTX−M*_*/sulII*	-	-	1	-	1
AR gene-negative isolates	1	-	-	-	1
Total	9	50	2	4	65
Virulence gene profiles					
* stx1*	-	14	-	1	15
* stx1/stx2*	-	2	-	-	2
* stx1/eae*	-	1	1	-	2
* eae*	1	3	-	-	4
Non-virulent isolates	8	30	1	3	42
Total	9	50	2	4	65

^a^Unknown phylo-group

**Table 6:** Pathotypes, virulence gene profiles, AR gene combinations, phenotypic AR patterns and phylo-groups of potential virulent *E. coli* isolates from horses

Pathotype	VG profile	AR gene profiles	Phenotypic AR patterns	Phylo-group	Sample code
STEC	*stx1*	*bla*_*TEM*_	-	B1	27 A
	*stx1*	*bla*_*TEM*_	-	B1	46 A
	*stx1*	*bla*_*TEM*_	-	U	52 A
	*stx1*	*bla*_*TEM*_	AMC	B1	45 A
	*stx1*	*bla*_*TEM*_	AMC	B1	47 A
	*stx1*	*bla*_*TEM*_	CRO	B1	40 A
	*stx1*	*bla*_*TEM*_	CRO	B1	41 A
	*stx1*	*bla*_*TEM*_	AK, S	B1	58 A
	*stx1*	*bla*_*TEM*_	CRO, AMC, AK	B1	39 A
	*stx1*	*bla*_*TEM*_	CRO, AMC	B1	28 A
	*stx1*	*bla*_*TEM*_, *sulII*	-	B1	53 A
	*stx1*	*bla*_*TEM*_, *sulII*	AMC, SXT	B1	56 A
	*stx1*	*bla*_*TEM*_	CRO, AMC, AK, CZ, S	B1	60 A
	*stx1*	*bla*_*TEM*_	GN, CRO, AMC, AK, CZ, S	B1	62 A
	*stx1*	*bla*_*TEM*_	CRO, SXT, AMC, AK, CZ, S	B1	57 A
	*stx1/stx2*	*bla*_*TEM*_, *bla*_*CTX−M*_	-	B1	42 A
	*stx1/stx2*	*bla*_*TEM*_	CRO	B1	68 A
	*stx1/eae*	*bla*_*TEM*_	-	D	29 A
	*stx1/eae*	*bla*_*TEM*_	CRO, AMC, CZ	B1	69 A
EPEC	*eae*	-	CRO, AMC	A	23 A
	*eae*	*bla*_*TEM*_	-	B1	30 A
	*eae*	*bla*_*TEM*_	-	B1	31 A
	*eae*	*bla*_*TEM*_	-	B1	32 A

## Discussion

In the current study, molecular pathotyping of equine *E. coli* isolates showed that more than one-third of them belonged to one of the diarrheagenic *E. coli* pathotypes including STEC and EPEC; the most prevalent pathotype was STEC (more than one-fourth of the isolates) followed by potential EPEC (less than 5 %). A few studies have reported the pathotypes in horses which mostly revealed low prevalence of them; Kennedy et al. (2018) in Ireland showed that none of the equine *E. coli* isolates obtained from 83 fecal samples were STEC or EPEC [18]. In the USA, a very low STEC prevalence, one from 242 equine *E. coli* isolates, has been reported [19]. Also Pichner et al. (2005) found only one STEC isolate among the 400 screened horse fecal samples in Germany [20]. Hamzeh et al. (2013) and Luna et al. (2018) have been observed STECs in 16.7 % and 11.7 % frequencies, respectively; the sample size in the two latter studies were small (less than 20 horses) [21, 22]. Chandran et al. (2013) have screened *E. coli* isolates from 11 different host sources and revealed that EPEC had the highest prevalence in horses (50 %) [23]. Despite the low STEC prevalence in horses, exposure to horse feces has been reported as a significant risk factor for clinical cases of STEC infections in humans [24, 25].

STEC pathotypes are usually detected from healthy horses and no clinical features have been observed in these cases. In a research in USA, positive horses for STEC were kept on farms containing ruminants [19]. Generally, ruminants are considered as the primary reservoir of the intestinal pathotypes; equids are not the main source of STEC and EPEC and they are known as spill-over hosts, the secondary species host exposed to the STEC through close contact with ruminants or feeding materials contaminated with ruminant manure [21]. Interestingly, all detected pathotypes of this study belonged to the horses with no history of ruminant direct contact.

Most STECs of this research were only positive for *stx1* gene and some possessed *stx1* and *stx2* simultaneously. It is believed that *stx1* and *stx2* positives could cause more severe cases of STEC infections in human [26, 27]. The STEC strains which are positive for *eae* gene could potentially induce the attaching and effacing lesions in human. It has to be considered that the detection of the *stx* and *eae* genes is not enough to determine the pathogenicity and virulence of the *E. coli* strains recovered from animals in the human. Therefore, further phenotypic evaluations in laboratory animal models and intestinal cell lines are considered essential.

The occurrence of antimicrobial resistant *E. coli* in companion animals has drawn attention as a public health issue in the last decade [28]. Resistance to highly prescribed antimicrobial agents such as betalactames, aminoglycosides and sulphonamides has been studied in equine *E. coli* strains [29].

Our phenotypic results revealed that the prevalence of all the resistance phenotypes were less than 50 %. The highest prevalence of antimicrobial resistance has been observed against amoxicillin/clavulanic acid followed by ceftriaxone. Penicillins, sulfonamides and aminoglycosides are amongst the most commonly used antimicrobial classes in equine medicine for various conditions such as respiratory, digestive and pyogenic infections [30]. In a study in South Africa, resistance rate to ceftriaxone and amikacin were similar to the ones found in this work, while the prevalence of resistant isolates to amoxicillin/clavulanic, trimethoprim/sulphamethoxazole and gentamicin were higher than the current study [31]. Fortunately, our resistance rate to trimethoprim/sulphamethoxazole was notably low while other studies worldwide reported higher frequencies. A wide range of resistance against streptomycin and gentamicin in *E. coli* equine isolates has been reported from various studies, explaining that MDR in this study is rather low (less than 15 %). Variation in prevalence of antimicrobial resistance may be due to evaluation method, sample size, season, antibiotic prescription patterns, microbial population type of gastrointestinal microflora and exposure to antimicrobial resistance determinants [32]. Multidrug resistant bacteria can lead to complicated infections in the susceptible hosts and are addressed as a major public health issue [33]. According to MDR definition, a multidrug resistant bacterium is non-susceptible to at least one antimicrobial agent from three or more different antibiotic categories [17]. In the current study, more than one-fourth of the *E. coli* isolates and a considerable number of VG-positives were resistant against multiple antibiotics which are highly prescribed in human and equine medicine. This is less than the reported MDR prevalence in Kennedy et al. (2018) and de Lagarde et al. (2020) studies [18, 34]. Dissemination of MDR bacteria may cause the spread of nosocomial and community-acquired infections which could lead to rising antibiotic use, healthcare costs, morbidity and mortality [35].

Two main mechanisms have been proved for MDR; accumulation of several resistance genes by the bacteria and increased expression of resistance genes [36]. The dissemination of antimicrobial resistance is mainly associated with AR genes which are mostly located on mobile genetic elements. Thus, detection of resistance genes in bacteria may help to understand the resistance transmission and improvement of antibiotic-therapy strategies. In this study *bla*_*TEM*_ was the most frequent AR gene, identified significantly higher (P < 0.05) than the other genes. Similarly, Johns et al. (2012) and Kennedy et al. (2018) reported high frequency of *bla*_*TEM*_ in UK and Ireland respectively, while Gharaibeh et al. (2020) detected the gene only in 15.5 % of equine *E. coli* isolates in Jordan [18, 37, 38].

The next most prevalent genes were associated with resistance against sulfonamides including *sulII* (10.7 %) and *sulI* (1.5 %) which were considerably lower than the 57 % prevalence reported by Kennedy et al. (2018) in Ireland [18]. For the remaining genes, our results are comparable with the study in Jordan; the prevalence of our screened genes were between 0 and 6 % while the frequencies in the Gharaibeh et al. (2020) study were more than 10 % [38]. The diversity in prevalence rates may be due to the use of different methods, genotypic variety of *E. coli* populations and antibiotic exposure in horse in different countries.

In the current study, phylogenetic assessment of the equine isolates showed no relationship among virulence, resistance and phylogenetic background; resistant/non-resistant and virulent/non-virulent *E. coli* strains frequently belonged to B1 phylo-group. In agreement with our results, many studies around the world have reported B1 as the predominant phylo-type in equine *E. coli* isolates [34, 39, 40]. Conversely, Sukmawinata et al. (2019), reported B2 as the most common phylogenetic group among extended-spectrum β-lactamase-producing *E. coli* isolates from healthy thoroughbred race horses in Japan [41]. Phylo-typing of *E. coli* strains help to determine the evolutionary relationships among the microorganisms and is a fundamental issue in microbial studies. High dissemination of B1 in all isolate types could be due to various reasons such as nutrition, host species, sex, age, body mass, climate, geographic location and the combination of gut microflora [42].

## Conclusions

In this study, a significant number of equine isolates belonged to one of the diarrheagenic *E. coli* pathotypes including STEC and potential EPEC, in which the most prevalent pathotype was STEC. Equids are known as spill-over hosts when exposed to the STEC through close contact with ruminants or feeding materials contaminated with ruminant manure. All detected pathotypes of this study belonged to the horses with no history of ruminant contact, which indicates that horses may take a role as a potential reservoir in spreading virulent pathotypes. Moreover, some VG-positive isolates were recognized as MDR. All the sampled animals in this study were riding horses with close contact to human including horse riders, club personnel and spectators. This represents the pathogenic and zoonotic potential of the equine strains in human medicine and would place emphasis on the design of antimicrobial resistance stewardship programs to control the dissemination of virulent and multi-drug resistant *E. coli* strains in the community.

## Data Availability

The datasets used and/or analyzed during the current study are available from the corresponding author on reasonable request.

## Abbreviations

AK: Amikacin
AMC: Amoxicillin/clavulanic acid
AMR: Antimicrobial resistance
*arpA*: Ankyrin repeat protein A
*chuA*: *E. coli* heme-utilization gene A
CRO: Ceftriaxone
CZ: Cefazolin
DAEC: Diffusely adherent *E. coli*
DEC: Diarrheagenic *E. coli*
*eae*: *Escherichia coli* attaching and effacing gene
EAEC: Enteroaggregative *E. coli*
EHEC: Enterohemorrhagic *E. coli*
EHECs: Enterohemorrhagic *Escherichia coli* strains
EIEC: Enteroinvasive *E. coli*
EPEC: Enteropathogenic *E. coli*
ETEC: Enterotoxigenic *E. coli*
GN: Gentamicin
PCR: Polymerase chain reaction
S: Streptomycin
SPSS: Statistical Package for the Social Sciences
STEC: Shiga toxin-producing *E. coli*
STECs: Shiga toxin-producing *Escherichia coli strains*
Stx: Shiga toxin
SXT: Trimethoprim-sulphamethoxazole
trpA: Tryptophan synthase alpha
TspE4.C2: An anonymous DNA fragment in *E. coli*
VG: Virulence gene
*yjaA*: *E. coli* K12 gene
